# Advanced Hodgkin Lymphoma: a New Era of Therapy

**DOI:** 10.4084/MJHID.2014.063

**Published:** 2014-09-05

**Authors:** Eldad J. Dann

**Affiliations:** 1 Department of Hematology and Bone Marrow Transplantation; 2 Blood Bank and Transfusion Service, Rambam Health Care Campus; 3 Bruce Rappaport Faculty of Medicine, Technion, Israel Institute of Technology

## Abstract

Therapy of advanced Hodgkin lymphoma (HL) is a rapidly changing field due to a lot of currently emerging data. Treatment approaches are presently based on either the Kairos principle of giving aggressive therapy upfront and considering de-escalation of therapy if the interim PET/CT is negative or the Chronos principle of starting with ABVD followed by escalation of therapy for patients with positive interim PET/CT. The International Prognostic Score (IPS) is still valid for decision-making regarding the type of initial therapy, since patients with a high score do have an inferior progression free survival (PFS) with ABVD compared to those with a low score. Escalated BEACOPP administered upfront improves PFS; however, increase in the overall survival (OS) has not been confirmed yet, and this therapy is accompanied by elevated toxicity and fertility impairment. Completion of ongoing and currently initiated trials could elucidate multiple issues related to the management of HL patients.

## Introduction

The treatment of advanced-stage Hodgkin lymphoma with the MOPP regimen (mechloroethamine, vincristine, procarbazine and prednisone) pioneered the use of chemotherapeutic protocols for therapy of malignancies. When the high rate of secondary leukemias was established, MOPP was replaced with the ABVD (adriamycin, bleomycin, vinblastine and dacarbazine) protocol developed by Bonadonna et al, which has been the standard of care since early 1970s.^1^ During the last 15 years a debate is ongoing as to whether a more intensive protocol such as escalated BEACOPP introduced by the German Hodgkin Study Group should be employed and for whom.^2^ The 10-year freedom from treatment failure and OS in the arm receiving 8 cycles of escalated BEACOPP were 82% and 87%, respectively.^3^ The IPS^4^ was re-evaluated by several study groups^5^,^6^ and proved to be an efficient tool in identifying the group of patients who had a reduced PFS if treated with ABVD. Recently, a 23-gene array was reported to be superior to the IPS and was suggested to become a potential predicting factor.^7^ Since patients with high IPS have an increased failure rate, the gene array could become a tool for upfront decision-making regarding treatment strategy in patients who may do well with a standard-dose therapy and in those who would require intensified therapy upfront.

Some phase II studies showed that therapy could be tailored based on interim imaging and IPS, thus saving escalated BEACOPP only to a limited subgroup of patients and preserving fertility in 88% of female patients.^8^–^10^ In the last decade, interim PET/CT performed following 2 cycles of therapy demonstrated a high negative predictive value which enables using less intensive and therefore less toxic regimens in patients with a negative interim scan. On the other hand, patients with a positive interim PET/CT following 2 cycles of ABVD are in a high risk of treatment failure, which necessitates further therapy escalation.^11^

The current ongoing studies are designed to minimize therapy for patients with low risk of disease progression in order to reduce toxicity and late side effects, such as secondary tumors, cardiac toxicity and loss of fertility. Present trials try to resolve the dispute between the Chronos principle of starting a standard low toxic regimen like ABVD and augmenting therapy only for patients with adverse predictive factors and the Kairos principle claiming that high efficiency highly toxic therapy should be started to all patients and only individuals with good prediction should have their therapy reduced.

This is a major issue, since the median age of patients with HL is 34 years and they have a life expectancy of another 50 years. Hence, joint international efforts are required to determine the optimal therapeutic strategies and spare these patients from late treatment-related adverse effects.

## Results of Therapy Using Current Protocols

Recently published randomized trials have demonstrated 5-year PFS of 74%–76% and OS of 88%–90% for patients with stage IIB, III and IV disease or IA, IIA bulky mediastinal mass treated with the ABVD regimen. Radiotherapy was administered to 41%–62% of these patients. (Table 1). A sub-analysis of the data according to the IPS demonstrated PFS of 77% and OS of 91% for patients with IPS 0–2 and PFS of 67% and OS of 84% for patients with IPS 3–7. The superiority of escalated BEACOPP in providing a higher PFS was demonstrated in several randomized control studies, although a statistically significant OS benefit was not achieved. The difference between the GHSG and the four other studies could be related to the total cohort size of 935 patients in the four studies and 2182 patients in GHSG HD15 trial (Table 1).^12^–^16^ Of interest, 6 cycles of escalated BEACOPP have been shown to result in a significantly better PFS than 8 cycles of escalated BEACOPP and an improved OS.^16^ However, the 10–15% difference in PFS observed in these patients, comes with impaired fertility is women and sterility in a vast majority of men, a heavy toll for this young population. A higher cumulative incidence ratio of secondary myelodysplastic syndrome (MDS), or secondary acute leukemia was reported in patients receiving ≥4 cycles of escalated BEACOPP compared to less than 4 cycles or other chemotherapy regimen (1.7%,0.7%,0.3%, respectively).^17^

## Use of Interim PET/CT for Tailoring Therapy in Advanced Hodgkin Lymphoma

Gallamini et al.^11^ demonstrated the capability of interim PET/CT to define the low-risk population that has a negative interim PET and carries a risk of relapse of 10% only, and the high-risk group that has a positive interim study and carries a risk of disease progression of 60–80% if treated with ABVD. Results of a retrospective international multicenter study demonstrated that negative predictive value of the interim PET-2 was around 95%, and PFS of patients with a positive interim PET was 28%.^18^ At same time, it was demonstrated that BEACOPP therapy could be tailored based on IPS and interim scintigraphy (from 1998–2001 with Gallium scan and from 2001–2005 with PET) providing both high PFS and OS, with reduction in toxicity and preservation of fertility in more than 85% of women.^9^,^19^ Several studies are currently ongoing using interim PET/CT for tailoring therapy. Of patients treated with 2 cycles of ABVD upfront, 80–85% have a negative interim PET/CT, and 15–20% have a positive scan defined as an uptake higher than in the liver. Escalation of therapy could salvage 60%–76% of these patients.^20^,^21^ A further follow-up is needed to evaluate if a high PFS is maintained in patients with negative interim PET/CT and whether radiation therapy could be omitted in patients treated with ABVD who had a bulky disease at diagnosis and a negative interim or end-of-therapy PET/CT.

## Ongoing Studies of Advanced Hodgkin lymphoma Therapy

Several large trials are ongoing or recently finished and waiting for a longer follow-up prior to publication (Table 2). These studies usually use interim PET/CT for tailoring therapy of individual patients. The RATHL (response adapted therapy in advanced Hodgkin lymphoma) study, initiated in the UK. has become a collaborative European trial. It recruited 1214 patients, 84% of whom had negative interim PET/CT and were further randomized to receive either 4 more cycles of ABVD or AVD (adriamycin, vinblastine, dacarbazine). Only 2.4% of these patients received radiation therapy. The 2-year PFS in the group with negative interim PET/CT was 86%. In the group of patients with positive interim PET (16% of patients) whose therapy was changed to escalated BEACOPP for 4 more cycles or BEACOPP-14, 76% had a negative PET/CT-3 that was performed 8–9 weeks after therapy intensification (escalated BEACOPP ×3). At a 1-year follow-up, 22% of these patients had disease progression or died.^21^

The US intergroup S-0816 study enrolled 357 patients with stage III, IV disease who received 2 cycles of ABVD followed by PET/CT. Eighty two percent of patients had negative interim study (Deauville score 1–3). These patients were treated with four additional courses of ABVD. The remaining 18% of patients had positive PET/CT (Deauville score 4–5) and were planned for 6 cycles of escalated BEACOPP; however, in 10% of these patients therapy was not escalated due to physician’s choice. The 1-year PFS for the patients with negative and positive PET/CT was 85% and 72%, respectively. The PFS for the whole group was 84% and OS – 98%.^22^

The Italian GITIL 0607 study registered 730 patients. Treatment of patients with IPS 0–7 included two cycles of ABVD followed by interim PET/CT. Patients with negative PET (82%) had a total of 6 cycles of ABVD followed by PET/CT. If the result of this imaging was negative, the patient was further randomized to either consolidative radiation therapy to the bulky mediastinal mass or no radiation. Patients with positive interim PET/CT (18%) were randomized to escalated BEACOPPx4 followed by standard BEACOPPx4 with or without rituximab. The 2-year PFS for all patients, those with interim negative and those with positive interim PET, was 81%, 85% and 61%, respectively.^23^

The Israeli H2 study recruited 180 patients with advanced HL. Patients with IPS 0–2 started therapy with two cycles of ABVD and those with IPS 3–7 received two cycles of escalated BEACOPP. An interim PET was performed following 2 cycles of treatment. If PET was negative, patients had four additional cycles of ABVD. If interim PET was positive, four cycles of escalated BEACOPP were administered. Eighty five percent of interim PET/CT scans were interpreted as negative and 15% as positive. Interim PET/CT was negative in 88% of patients with IPS 0–2 and 80% of patients with IPS 3–7. Chemotherapy was de-escalated in 89% of patients with IPS 3–7 and at 3 years only 13% of the whole group progressed. At a median follow-up of 26 months the 3-year PFS was 85%.^24^

The issue of early salvage therapy for patients with advanced HL who had a positive interim PET/CT following two cycles of ABVD was assessed by the Italian Lymphoma Study Group in the HD0801 study that enrolled 520 patients. Patients with negative interim PET/CT received a total of 6 cycles of ABVD and were further randomized to radiation therapy to bulky mediastinal masses or to no radiation therapy. Patients with positive interim PET/C were treated with salvage protocol including four cycles of IGEV (ifosfamide, gemcitabine, etoposide, vinorelbine and prednisolone). After salvage therapy a further assessment was performed. Patients with negative PET (58% of individuals) underwent autologous stem cell transplant with the BEAM (carmustine, etoposide cytarabine, and melphalan) protocol. Following four cycles of IGEV, patients with positive PET/CT (42%) underwent tandem autologous transplantation or autologous followed by allogeneic transplantation if a matched related donor was available. The majority of patients with positive interim PET could be salvaged with an early shift to high-dose chemotherapy and stem cell rescue. The reported 2-year PFS and OS based on PET/CT results following 2 cycles of ABVD was 75.7% and 98.6% for patients with negative interim study and 64.1% and 86.3%, respectively for those with positive interim scan. Eighty nine percent of patients treated with this salvage protocol had a 2-year relapse free survival.^25^,^26^

Several ongoing studies use 2 cycles of escalated BEACOPP for patients with IPS 0–7 followed by interim PET/CT. In the HD18 trial by the German Hodgkin Study Group, patients with negative interim PET/CT are randomized to additional 2 or 6 cycles of escalated BEACOPP, while patients with positive interim PET/CT are treated with 6 extra cycles of escalated BEACOPP. In this study, radiation therapy is applied only to patients who had residual positive uptake sites at the end of treatment.^27^

The French LYSA AHL2011 study randomizes patients with IPS 0–7 to the standard arm where 6 cycles of escalated BEACOPP are used regardless of interim PET/CT performed after 2 cycles, and to the experimental arm where patients with negative interim PET post 2 cycles of escalated BEACOPP have their therapy de-escalated to 4 cycles of ABVD, while patients with a positive interim scan receive additional 4 cycles of escalated BEACOPP (a total of 6 cycles).^28^

The trials discussed above are only part of ongoing studies using interim PET as a predictive value for further therapy of individual patients. Recently initiated EORTC H11 is checking the predictive accuracy of early interim PET performed following a single cycle of escalated BEACOPP. All patients in observational arm are treated with 6 cycles of therapy, which remains unchanged irrespective of interim PET results, while in the experimental arm therapy is de-escalated to ABVD if interim PET is negative. This interesting study could potentially elucidate some challenging issues associated with early use of interim PET following escalated BEACOPP and reduction of therapy, which is expected to maintain fertility in young female patients.^29^,^30^ Further studies have been lately initiated using the anti-CD30 antibody-drug conjugate as part of first-line chemotherapy including both ABVD type protocols and escalated BEACOPP. These regimens have been modified to exclude bleomycin and oncovin due to their major lung and neurotoxicity.

## Discussion

The treatment of Hodgkin lymphoma is a rapidly evolving area. Many ongoing studies are designed to establish the role of upfront intensive therapy, which presents the Kairos principle, employing escalated BEACOPP, followed by de-escalation of therapy in patients with negative interim PET/CT. Other studies use the Chronos principle of starting with ABVD and further therapy escalation only for patients with positive interim PET/CT, (Deauville score 4,5) which clearly indicates their high risk for disease progression. The latter trials apply various escalation regimens ranging from escalated BEACOPP to a salvage protocol including 4 cycles of IGEV followed by stem cell collection and autologous transplantation. While all these studies are expected to provide PFS and OS rates after 4–5 years of follow-up, long-term outcomes (up to 15 years) including late side effects and problems like fertility, fatigue, secondary malignancy, ischemic and valvular heart disease, are not less important. These issues are crucial for HL patients, given their young age at diagnosis and a life expectancy of 50 years ahead of them, if appropriately treated.

## Conclusion

Patients with advanced HL and IPS 0–2 can be initially treated with ABVD; however, these patients should undergo interim PET/CT following 2 cycles of therapy. Patients with negative interim PET/CT can safely continue with additional 4 cycles of ABVD. If an interim scan is positive, the data from retrospective studies suggest a low PFS of 12–27% only; however, escalation of therapy led to remission in 65% of patients. To date, only preliminary data from currently ongoing GITIL HD0607 study are available; nevertheless, it has already been demonstrated that patients with positive PET-2 achieved a 2-year FFS of 67% after escalation of their therapy. Moreover, preliminary data from prospective studies like RATHL show that patients in whom therapy was escalated based on a positive interim PET-2 achieved a complete response at the end of chemotherapy in 76% of cases. The information available suggests that therapy intensification is beneficial; however, a stronger conclusion will be drawn when the results of these 2 studies are published. Patients with advanced disease and IPS ≥3 are in a higher risk of treatment failure if therapy is initiated with ABVD than those in whom therapy with a more intensive regimen (escalated BEACOPP) is started. This cohort should also undergo interim PET/CT after 2 cycles of therapy. Patients with positive interim PET/CT are in a high risk group and need to continue with the intensified therapy. Patients with negative interim PET/CT could do as well with reduction of therapy to a standard regimen (ABVD) if they initiated with escalated BEACOPP or proceed with ABVD if this was their original therapy. However, reduction of therapy from escalated BEACOPP to ABVD in this subgroup of patients is yet experimental awaiting confirmation by the ongoing studies.

Radiation therapy can be omitted for patients that were treated with 6 cycles of escalated BEACOPP and have a residual mass at the end of therapy which is PET/CT negative. This conclusion is based on the results of the HD15 study and is the current approach applied in the ongoing HD18, both by the German Hodgkin Study Group. In the RATHL study, less than 5% of patients with negative interim PET received radiation therapy. The 12-month PFS was promising, but the final verdict will be available when the results of the study are published.

The effect of adding brentuximab vedotin to the chemotherapy regimen for patients with positive interim PET/CT will need assessment by an international collaborative study.

## Figures and Tables

**Table 1:** Randomized trials comparing ABVD, Stanford V and BEACOPP regimens

Study	Disease stage	Pts No	Protocol	Radiation therapy	PFS %	OS %

***National Cancer Research Institute*** (NCRI), UK ISRCTN 64141244^31^	Ib,IIb,III,IV or bulky IA,IIA	520	ABVD	53%	76 5Y	90 5Y
			Stanford V	73%	74	92

Intergruppo Italiano Linfomi^32^	IIb,III,IV	355	ABVD	62%	78 5Y	88 5Y
			Stanford V	66%	54	77

***Eastern Cooperative Oncology Group*** (ECOG) E2496^33^	Ib,IIb,III,IV or bulky IA,IIA	854	ABVD	41%	74 5Y	88 5Y
					IPS 0–2 77	IPS 0–2 91
					IPS 3–7 67	IPS 3–7 84
			Stanford V	75%	71	88 5Y
					IPS 0–2 78	IPS 0–2 93
					IPS 3–7 57*	IPS 3–7 77

Intergruppo Italiano Linfomi^12^	IIb,III,IV	331	ABVD BEACOPP esc ×8	66%	73	84 (NS)
				67%	85*	89

German Hodgkin Study Group HD15^16^	IIb,III,IV	2182	BEACOPP esc × 8	11%	84	92
			BEACOPP esc × 6		89*	95
			BEACOPP-14		85	94

German Hodgkin Study Group HD12^34^,^35^	III,IV	1670	BEACOPP esc ×8 BEACOPP (esc ×4 +base ×4)	Randomized 16% v 74%	86.4 5Y	92 5Y
					84.8 5Y	90.3 5Y

Gruppo Italiano Per Lo Studio Dei Linfomi HD 2000^14^	IIb,III,IV	103	ABVD BEACOPP (esc ×4 +base ×2)	46%	68 5-Y*	84 (NS)
		102		45%	81	92

European Organisation for Research and Treatment of Cancer (EORTC) 20012^13^	III,IV (IPS 0–3)	249	ABVDx8 BEACOPP (esc ×4 +base ×4)	No RT	73 4-y*	87 4-y (NS)
					83 4-y	90 4-y

Lymphoma Study Association (LYSA) H34^15^	III,IV (IPS 0–2)	150	ABVDx8 BEACOPP (esc ×4 + base ×4)	No RT	75 5-y*	92 (NS)
					93	99

* - Statistical significant.

**Table 2:** Trials for Patients with Advanced Hodgkin’s Lymphoma

Study and Risk Group	Treatment
**FIL HD0801** n=520^25^,^26^	
Stage IIb–IV (IPS 0–7)	2×ABVD followed by PET: if PET is negative, 4 × ABVD, randomize ±RT to bulky site if PET is positive, 4 × IGEV PET/CT If - AUTO BMT (BEAM ) If+ PAM-AUTO+ alloBMT or auto-BEAM
**LYSA AHL 2011** n=810^28^	
IIB, III, IV Standard arm	2xEB followed by PET2,regardless of results EBx2 PET-4 If neg EB ×2 IF+ salvage
Experimental arm	2 × EB followed by PET: if PET is negative, 4 xABVD if PET is positive 4 xEB
**Israeli H2 protocol** n=182^24^	
Stage IIb–IV (IPS 0–2) Standard risk	2×ABVD followed by PET: if PET is negative, 4 × ABVD, no RT; if PET is positive, 4 × EB + INRT to the PET positive site
Stage IIb–IV (IPS ≥ 3) High risk	2× EB followed by PET: if PET is negative, 4xABVD; if PET is positive 4 xEB +INRT to the PET positive site
**American Intergroup Study** (S0816)^22^	
	2×ABVD followed by PET: if PET is negative, 4 × ABVD, no RT; if PET positive and HIV is negative, 6 × EB, no RT; if PET is positive and HIV is positive, 6 × SB, no RT
**German Hodgkin Study Group** (HD18) n=1500^27^	
IIB bulky,III, IV (IPS 0–7) Standard arm	2×EB followed by PET: 6 × EB regardless of PET results
Experimental arm	2×EB followed by PET: if PET is negative, 2 × EB; if PET is positive, randomize to 6 × EB RT to residual nodes ≥ 2.5 cm
**GITIL HD0607** for patients with IIA Bulky n=730^23^	
stage IIB–IVB Interim positive PET/CT arm	2 × ABVD followed by PET: if PET is positive, randomize to: Arm A (4 × EB, repeat PET, if negative – 4 × SB) Arm B (4× R-EB, repeat PET, if negative 4 × R-SB) If PET is positive post 6 chemotherapy cycles, then high-dose chemo + auto BMT × 2 or auto BMT followed by allo BMT
Interim negative PET/CT arm	2 × ABVD followed by PET: if PET is negative, 4 × ABVD, then another PET: if negative, randomize to RT or no RT arm If second PET is positive, perform biopsy; if positive, 4 × salvage and auto BMT
**United Kingdom NCRI Lymphoma Study Group n=1214 patients**^21^	
stage IIB–IV and IIA with bulk or ≥3 sites Interim negative PET/CT arm	Baseline PET: 2×ABVD, then interim PET – if negative, randomize to 4×ABVD; or 4 × AVD
Interim positive PET/CT arm	Baseline PET: 2×ABVD, then interim PET: if positive, 4× BEACOPP-14, reassess with PET/CT; if negative, 2 more BEACOPP -14 if PET/CT is positive, salvage therapy or RT
**European Organization for Research on the Treatment of Cancer (EORTC) (H11) n=570**^29^	
Standard arm	1 × EB followed by PET/CT: 3 × EB regardless of PET results Patients with CR/PR on CT scan are treated with 2 × EB 36Gy RT to PET positive residual mass at the end of therapy
Experimental arm	1 × ABVD followed by PET/CT: if PET is positive, 3 × EB. Patients with CR/PR on CT scan receive 3 × EB if PET is negative, 3 × ABVD. Patients with CR/PR on CT scan 2 × ABVD 36Gy RT to PET positive residual masses at the end of therapy
**Millennium global study C25003 n=1040**^36^	
Standard arm	ABVDx2 PET/CT -(Deauville 1–4) ABVDx4 +(Deauville 5) off study
Experimental arm	(AVD+Brentuximab Vedotin)x2 PET/CT -(Deauville 1–4) receive(AVD+BV)x4 +(Deauville 5) off study

EB – escalated BEACOPP, SB – standard BEACOPP, R – Rituximab, IPS – International Prognostic Score, CR – complete remission, PR – partial remission, RT – radiation therapy; auto BMT – autologous bone marrow transplant, allo BMT – allogeneic bone marrow transplant, AVD – adriamycin, vinblastine, dacarbazine. Reproduced with permission from: Dann EJ, Curr Oncol Rep. 2012 Oct;14(5):403-10.
